# Incidence and predictors of surgical site infection after ORIF in calcaneus fractures, a retrospective cohort study

**DOI:** 10.1186/s13018-018-1003-y

**Published:** 2018-11-20

**Authors:** Hui Wang, Honglei Pei, Meiyun Chen, He Wang

**Affiliations:** 1 Department of Orthopaedic Surgery, The First Hospital of Hebei Medical University, Shijiazhuang, Hebei 050051 People’s Republic of China; 2 Department of General practice, The Second Hospital of Hebei Medical University, Shijiazhuang, Hebei 050051 People’s Republic of China; 3 Basic Medical College, Hebei Medical University, Shijiazhuang, Hebei 050000 People’s Republic of China

**Keywords:** Calcaneus fracture, Surgical site infection, Incidence, Risk factors, Hypothermia

## Abstract

**Background:**

Occurrence of calcaneus fractures is on the up trend. Owing to its unique anatomical morphology and limited soft-tissue envelope, management of calcaneus fractures is a challenge to the orthopaedic surgeon, and surgical site infection (SSI) is one of the serious postoperative complications. In order to decrease the incidence of wound breakdown and improve clinical outcomes, it is necessary to understand which factors were associated with SSI. The aim of this study was to identify predictors of SSI and quantify the incidence of SSI in calcaneus fractures following open reduction and internal fixation (ORIF).

**Methods:**

This retrospective study was performed at a level 1 trauma center from January 2014 to June in 2017. Data of adult patients with calcaneus fractures treated by ORIF were extracted from the electronic medical records. A total of 681 patients were collected. We reviewed the patients’ demographics, characteristics of fracture, treatment-related variables, and indexes of laboratory examination. Univariate and multivariate logistic analysis models were performed respectively to determine independent predictors of SSI.

**Results:**

Sixty-six patients developed SSI in this study. The overall incidence of SSI after ORIF of calcaneus fracture was 9.7%, with 2.9% for deep infection and 6.8% for superficial SSI. Independent predictors of SSI identified by multivariate analysis were open fracture (odds ratio = 9.48, 95% CI = 4.53–19.85, *P* = 0.00007), high-energy injury (odds ratio = 2.07, 95% CI = 1.16–3.70, *P* = 0.01437), ASA class 3 or higher (odds ratio = 3.50, 95% CI = 1.18–10.37, *P* = 0.02401), and intraoperative temperature < 36.0 °C (odds ratio = 1.69, 95% CI = 1.13–2.28, *P* = 0.04410).

**Conclusion:**

The SSI incidence was high (9.7%) for calcaneus fractures following ORIF. External fixation plays an important role in the treatment of severely displaced and depressed intra-articular or open calcaneus fractures. Increased ASA class and intraoperative hypothermia were associated with wound breakdown, and elaborative evaluation of fracture and soft-tissue damage was vitally necessary in this at-risk population.

## Introduction

Calcaneus fractures are commonly seen injuries in the fracture of foot. Previous studies have stated that calcaneus factures accounted for 1 to 4% of all adult fractures [1, 2], and 60–80% are displaced intra-articular calcaneus fractures (DIACFs) [1–4]. Calcaneus fractures especially DIACFs were mainly caused by an axial loading mechanism, which is directed through the somewhat laterally situated calcaneal tuberosity. Current data of clinical epidemiology of orthopedic trauma demonstrated proportion of traumatic fractures caused by traffic accident, fall from heights, and crushing injury were 20.4, 9.2, and 9.7%, respectively [5]. Approximately 30% of such fractures occur in manual laborers, and 20% are work related. In recent years, the optimal management of displaced intra-articular calcaneus fractures is controversial [6]. Open reduction and internal fixation is the optimum treatment strategies for severely displaced or depressed intra-articular and the open calcaneus fractures. The surgical treatment for calcaneus fractures aims to restore the heel height and length, realign the posterior facet of the subtalar joint, and restore mechanical axis of the hindfoot [7]. However, complications following surgical treatment are inevitable and surgical site infection (SSI) is a serious one. The prevalence of wound infection in closed calcaneus fractures varies from 2 to 25% [8–10]. Incidence of deep tissue infection following open calcaneus fractures has been reported up to 10–39%, and eventual amputation to 8.0–14% [11–13]. Debridement or implant removal and even amputation is needed to control severe wound infection, which will greatly affect these patients’ quality of life. Furthermore, readmission to hospital will substantially increases healthcare costs for patients and society. How to prevent the infection of surgical sites has become a major challenge for orthopedic surgeons.

Risk factors of postoperative wound infection following open reduction and internal fixation (ORIF) of calcaneus fractures have been identified by some previous studies, including male sex, a history of active smoking, diabetes mellitus, higher body mass index (BMI ≥ 30 kg/m^2^), drug abuse, open fracture, Sander type, delaying definitive fixation more than 14 days, operation time of more than 2 h, tourniquet time of more than 1.5 h, estimated blood loss, single-layered closure, surgeon experience, hospital stay, and high number of persons present in the operating room during entire surgical procedure [6, 8, 14–17]. However, most of these investigations possess the inherent features of data aging and small sample size and some literatures even suggest the opposite. We wished to ascertain whether there were relationship between these variables and wound infection by this large-simple study.

Given that, we designed this retrospective study to address this tissue. The purposes of this study were to [1] quantify the incidence of postoperative surgical site infection and [2] identify the independent risk factors associated with SSI treated by ORIF of calcaneus fractures.

## Patients and methods

Our investigation retrospectively identified patients 18 years or older with acute calcaneus fractures treated by ORIF at our hospital during January 2014 to June in 2017, with postoperative follow-up ranged from 12 to 42 months. Patients with complete follow-up data could be included in this study. The exclusion criteria were patients younger than 18 years, implant removal following calcaneus fractures, non-surgical treatment, pathological fractures, and old fractures (> 21 days from initial injury). Therefore, a total of 681 patients were finally included and related data were analyzed.

All data were abstracted from the electronic medical record. Demographic information of patients included gender, age, living places (rural or urban), occupation, height, and weight. BMI was divided into six groups according to the Chinese reference criteria: underweight, < 18.5; normal, 18.5–23.9; overweight, 24–27.9; obesity, 28–31.9; morbid obesity, 32 and more. Tobacco and alcohol consumption, drug addict and comorbidities (coronary heart disease, hypertension, diabetes mellitus, anemia) were also inquired and recorded.

**Fig. 1:**
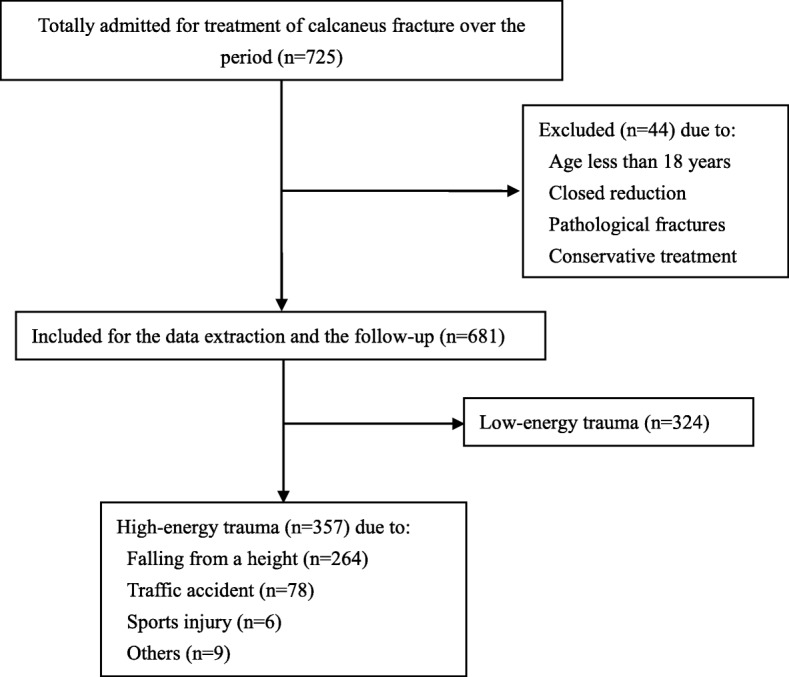
The etiology data of injury mechanism of the study group

Characteristics of fractures included Sander’s classification, injury mechanism, injury type (closed or open), side involved, and coexisting injuries. Injury mechanism was classified as high energy if the fractures caused by traffic accidents or fall from heights and sporting activity (Fig. 1).

Other treatment-related variables such as surgeon level (visiting and resident staff), preoperative stay, operation seasons, surgical approach (extended lateral approach, minimally invasive approach, and others), lengths of operative incision, operative time, intraoperative blood loss, intraoperative temperature (30 min before the end of operative procedure), fixation type, oral or intravenous use of antibiotic, and postoperative drainage tube were also extracted. Preoperative stay was defined as the interval between injury occurrence and operation and was divided into two groups: (1) ≤ 14 days and (2) > 14 days. It should be pointed out that we add up all incisions to calculate the exact lengths if there were several operative incisions in one approach. According to our protocol, antibiotics were given within 30 min before surgery as well as supplemental use of antibiotics due to prolonged operative time and obesity of patients after operation was identified as intraoperative use. ASA (American Society of Anesthesiologists) index [18] was used to evaluate patients’ physical status and surgical risk.

For preoperative variables of laboratory examination, we reviewed white blood cell (WBC), neutrophile granulocyte (NEUT), lymphocyte (LYM), monocyte (MON), basophilic granulocyte (BAS), serum total protein (TP), albumin (ALB), globulin (GLOB), and albumin/globulin value (A/G). According to their values, we divided these variables into normal (a range), above normal, and below normal.

Regarding to polytrauma, we defined it as injury more than one of the musculoskeletal, abdominal, cardiothoracic, urogenital, vascular, and central nervous systems. In order to quantify patients’ coexisting trauma by digits, we used “1” to represent the injury of different sites. For example, if a patient suffered from left calcaneus fracture of Sanders III, accompanied by 23-A3.1 fractures (according to the OTA/OA classification) of distal ulna and radius and traumatic rupture of the spleen, we label him as “2.”

SSI was defined based on the standard Center for Disease Control (CDC) definitions. Infections involving deep soft tissue, muscle, or fascia; persistent wound discharge or dehiscence; visible abscess or gangrenosis that requiring surgical debridement and implant exchange or removal were identified deep SSI. We diagnosed the infection as superficial SSI if it met at least one of the following criteria: only shin or subcutaneous at the wound site associated with infection and oral or intravenous antibiotics treatment for incision problems (redness, swelling, pain) but did not meet the criteria of deep SSI regardless of bacterial culture results.

This study was approved by the Institutional Review Board of our hospitals.

### Statistical analysis

A univariate logistic analysis was used to assess the relationship between each categorical variable and SSI. Mann-Whitney *U* test was used for non-normally distributed continuous variables and *t* test for normally distributed variables. The significance was set at *P* < 0.05 (*P < 0.05, **P < 0.01, ***P < 0.001, ****P < 0.0001). Variables tested to be approximately predictive (*P* < 0.1) of SSI by univariate analysis were entered into a multivariable logistic regression model to determine independent predictors of SSI. Adjusted odds ratios and their respective 95% CIs were reported in the multivariable analysis. The Hosmer–Lemeshow test was used to examine goodness-of-fit of this model, and the significance threshold was set at *P* > 0.05 indicated an acceptable fitness.

## Results

### Feature of the study sample

Overall, 725 patients with calcaneus fracture were collected. Six fractures were treated non-surgically; 27 patients were excluded because of closed reduction and percutaneous Kirschner wire fixation; 2 were pathological fractures and 9 patients were under 18 years; therefore, a total of 681 patients were treated by ORIF and included in this study. Of them, 625 were males and 56 were females, with a mean age of 42.4 years (range from 18 to 74) and male-to-female ratio of 11.2:1. Left side of calcaneus fractures were involved in 291 case and right side in 265, and 125 patients suffered from bilateral calcaneal fractures. There were 22 cases of Sanders grade I, 355 cases of Sanders grade II, and 210 and 94 cases of Sanders grade III and IV, respectively. One hundred sixty-two of the patients used tobacco productions. Thirty-five fractures were open, and all patients underwent debridement before ORIF. Of the open fractures, 16 patients developed SSI after surgery. The average time to definitive internal fixation was 5.7 days, with a minimum of 0 day and a maximum of 37 days. Three hundred fifty-seven patients with calcaneus fractures were caused by high-energy trauma. There were 264 falls from height, 78 vehicle collisions, 6 sporting injury, and 9 injured by others. The 681 patients sustained a total of 928 traumatic injuries associated with musculoskeletal, cardiothoracic, and other systems.

### Characteristics of SSI

There were 66 cases of SSI after ORIF of calcaneus fractures, with an incidence of 9.7%. Twenty (2.9%) patients experienced postoperative deep SSI, and 46 (6.8%) of the 66 cases developed superficial infection. The earliest diagnosis of SSI occurred at 3 days after operation, and the latest presentation was at 107 days, with a median time of 5 days. Fourteen cases of deep SSI and six superficial SSI of the infected patients’ bacterial culture results were extracted from the electronic medical record. *Staphylococcus aureus*, the most commonly seen pathogen, were cultured in 13 patients’ swab of wound secretions. Four were caused by *Escherichia coli*. *Pseudomonas aeruginosa* were identified in 1 case following Methicillin-resistant *Staphylococcus aureus* (MRSA) in 2 cases. Seventeen patients underwent irrigation and debridement to control the infection. Internal implant was removed in 2 cases because of persistent positive culture and apparent clinical signs of infection even after antibiotics treatments.

Demographical and perioperative variables such as age (41.4 versus 42.5 years, *P* = 0.43910), preoperative stay (5.7 versus 5.7 days, *P* = 0.97616), lengths of surgical incision (9.2 versus 9.0, *P* = 0.76514), and operative duration (117.4 versus 106.7 min, *P* = 0.05639) were presented in Table 1. However, there were no significant differences for these variables mentioned above between patients with and without SSI. The mean intraoperative blood loss was 155.6 ml for patients with a wound infection and 122.2 ml for the non-SSI group (*P* = 0.01501). The mean final intraoperative temperature was 35.8 °C in the group who developed SSIs compared with 36.3 °C in the group without SSIs (*P* = 0.03728). Surgical wound infection prolonged a mean of 14.7 days of hospitalization than that of non-SSI (31.0 versus 16.3 days, *P* = 0.00701), (Table 1).

**Table 1:** Continuous variables tested in patients with and without SSI

Variables	Patients with SSI (mean, median, range) (*n* = 66)	Patients without SSI (mean, median, range) (*n* = 615)	*P* value
Age (years)	41.4, 39 (18–66)	42.5, 43 (18–74)	0.43910
Hospital stay (days)	31.0, 21.5 (7–263)	16.3, 15 (4–76)	0.00701**
Preoperative stay (days)	5.7, 5 (0–27)	5.7, 5 (0–37)	0.97616
Operation duration (min)	117.4, 120 (35–250)	106.7, 95 (30–335)	0.05639
Intraoperative blood loss (ml)	155.6, 100 (10–1000)	122.2, 100 (10–800)	0.01501*
Lengths of incision (cm)	9.2, 8.5 (0.5–20)	9.0, 10 (0.5–27)	0.76514
Intraoperative temperature (°C)	35.8, 36.2 (34.8–36.9)	36.3, 36.2 (35.1–37.2)	0.03728*

Significant Variables (*P < 0.05; **P < 0.01; ****P < 0.0001)

### Risk factors of SSI

Factors that increased the risk of SSI in univariate analysis are summarized in Table 2. These predictors included injury mechanism (high-energy injury), open fractures, preoperative use of antibiotics, ASA score ≥ 3, operative duration, intraoperative temperature < 36 °C, coronary heart disease, and preoperative basophilic granulocyte (BAS) count. Other variables of demographical characteristics, underlying diseases, biochemical tests, and perioperative factors were not associated with the incidence of SSI (Table 2). All these approximately predictive variables were entered into the multivariate logistic regression model to determine the significant ones. After adjustment for confounding factors, injury mechanism (high-energy injury) (*P* = 0.01437), open fracture (*P* = 0.00007), ASA ≥ 3 (*P* = 0.02401), and intraoperative temperature < 36.0 °C, (*P* = 0.04410) remained statistically significant for the occurrence of SSI, with the adjusted odds ratio was 2.07 (1.16–3.70), 9.48 (4.53–19.85), 3.50 (1.18–10.37), and 1.69 (1.13–2.28), respectively. However, coronary heart disease, operative duration, basophilic granulocyte (BAS) count, and preoperative use of antibiotics were eliminated from the multivariate analysis as independent risk factors of SSI (Table 3). The value of Hosmer–Lemeshow test demonstrated the preferable fitness (*X*^2^ = 3.303, *P* = 0.347).

**Table 2:** Univariate analysis of risk factors associated with SSI

Variables	SSI (*n* = 66, 9.7%)	No SSI (*n* = 615, 90.3%)	*P* value
Open fracture	16 (24.2)	19 (3.1)	0.00003****
Sanders grade			0.08191
I	4 (6.1)	18 (2.9)	
II	36 (54.5)	319 (51.9)	
III	19 (28.8)	191 (31.1)	
IV	7 (10.6)	87 (14.1)	
Operation season (summer)	28 (42.4)	233 (37.9)	0.78615
Intraoperative blood loss (≥ 400 ml)	2 (3.0)	15 (2.4)	0.77030
Extended lateral approach	15 (22.7)	181 (29.4)	0.24816
Fixation type (plate and screws)	37 (56.1)	294 (47.8)	0.20429
Length of operative incision (cm)			0.51307
< 7	23 (34.8)	200 (32.5)	
7–11	24 (36.4)	210 (34.1)	
> 11	19 (28.8)	205 (33.3)	
Operative time			0.05660
< 120 min	29 (43.9)	380 (61.8)	
120–180 min	36 (54.5)	205 (33.3)	
> 180 min	1 (1.5)	30 (4.9)	
Intraoperative temperature(< 36.0 °C)	21 (31.8)	103 (16.7)	0.01807*
Gender			0.26118
Male	63 (95.5)	562 (91.4)	
Female	3 (4.5)	53 (8.6)	
Preoperative stay			0.36943
≤ 14 days	63 (95.5)	599 (97.4)	
> 14 days	3 (4.5)	16 (2.6)	
Surgeon level (resident and visiting staff)	16 (24.2)	111 (18.0)	0.22901
Injury mechanism (high energy)	47 (71.2)	310 (50.4)	0.00204**
Obesity (BMI ≥ 28.0)	10 (15.2)	71 (11.5)	0.39142
Diabetes mellitus	0 (0)	20 (3.3)	0.99836
Hypertension	7 (10.6)	53 (8.6)	0.58909
Coronary heart disease	3 (45.5)	8 (1.3)	0.06311
History of anemia	0 (0)	3 (0.5)	0.99910
Tobacco consumption	13 (19.7)	149 (24.2)	0.41327
History of alcohol intake	12 (18.2)	117 (19.0)	0.86844
Allergic history	4 (6.1)	25 (4.1)	0.44905
Previous operation	9 (13.6)	49 (8.0)	0.12219
ASA score (≥ 3)	5 (7.6)	14 (2.3)	0.01903*
General anesthesia	11 (16.7)	64 (10.4)	0.67229
Preoperative antibiotics use	17 (25.8)	69 (11.2)	0.00107**
Intraoperative antibiotics use	61 (92.4)	555 (90.2)	0.56822
Drainage use	39 (59.1)	332 (54.0)	0.42941
TP (< 65 g/L)	28 (42.4)	221 (36.0)	0.29930
ALB (< 40 g/L)	19 (28.8)	137 (22.3)	0.23307
GLOB (< 20 g/L)	15 (22.7)	91 (14.8)	0.16344
A/G			0.46406
References (1.2–2.4)	59 (89.4)	564 (91.7)	
< 1.2	1 (1.5)	11 (1.8)	
> 2.4	6 (9.1)	40 (6.5)	
WBC (10^9^/L)			0.46511
References (4–10)	47 (71.2)	465 (75.6)	
< 4	1 (1.5)	5 (0.8)	
> 10	18 (27.3)	145 (23.6)	
NEUT (10^9^/L)			0.39405
References (1.8–6.3)	39 (59.1)	394 (64.1)	
> 6.3	27 (40.9)	217 (35.3)	
LYM (10^9^/L)			0.99515
References (1.1–3.2)	56 (84.9)	533 (86.7)	
< 1.1	10 (15.2)	71 (11.5)	
> 3.2	0 (0)	11 (1.8)	
MON (10^9^/L)			0.79901
References (0.1–0.6)	31 (47.0)	298 (48.5)	
> 0.6	35 (53.0)	315 (51.2)	
BAS (10^9^/L)			0.08710
References (0–0.06)	60 (90.9)	577 (93.8)	
> 0.06	6 (9.1)	18 (2.9)	

*TP* total protein, *A* albumin, *G* globulin, *A/G* albumin/globulin, *WBC* white blood cell, *NEUT* neutrophile, *LYM* lymphocyte, *MON* monocyte, *BAS* basophilic

Significant Variables (*P < 0.05; **P < 0.01; ****P < 0.0001)

**Table 3:** Variables tested for multivariate analysis

Variable	*P* value	Odds ratio	95% CI
Open fracture	0.00007****	9.48	4.53–19.85
High-energy injury	0.01437*	2.07	1.16–3.70
ASA ≥ 3	0.02401*	3.50	1.18–10.37
Operative duration	0.08287	1.47	0.95–2.26
Intraoperative temperature < 36.0 °C	0.04410*	1.69	1.13-2.28

*CI* Confidential interval

Significant Variables (*P < 0.05; ****P < 0.0001)

## Discussion

With the rapid development of construction and transportation industries, calcaneus fractures actually saw an uptick in its occurrence [1, 2]. Management of calcaneus fractures is a challenge to the trauma surgeon on account of its unique anatomical morphology and limited soft-tissue envelope. However, the benefits of operative treatment may be offset by the subsequent complications. Wound infection, one of the most serious complications, can lead to implant removal, nonunion, and even amputation. From an economic perspective, SSI account for 17% of nosocomial infections, and cost between 1 and 10 billion US dollars annually [19]. All of these endpoint events will inevitably affect the quality of patients’ life. In order to decrease the incidence of wound breakdown and improve clinical outcomes, it is necessary to understand which factors were associated with these complications. In this study, the overall SSI rate was 9.7% with 2.9% for deep infection and 6.8% for superficial infection. SSI prolonged the hospital stays up to 14.8 days. Open fractures, high-energy injury, and ASA score ≥ 3 were identified as independent predictors of the occurrence of SSI.

The overall prevalence of SSI in our study was 9.7%, which was conformed to previously reported data [16, 20] and lower than Folk JW’s and Liang Ding’s retrospective studies [14, 21]. Incidence of SSI after open calcaneus fractures in this study was 45.7% (16/35), which was rather high compared to reported data in previous studies [12, 22]. Specifically, to point out, our hospital was the tertiary referral center and the patients were likely presenting with more severe injuries and more difficult to treat (357 high-energy injury in 681 calcaneus fractures; 681 patients sustained a total of 928 traumatic polytrauma). All these factors together may lead to a higher prevalence of infection in open fracture. Incidence of wound infection in closed calcaneus fracture was 7.7% (50/646) in our study, and this value was in the range of reported data (2–25%) [8–10]. According to the results of this investigation, occurrence rate of superficial and deep infection was 6.8 and 2.9%, respectively. The prevalence of deep infection has been quoted as being between 1.8 and 21% [16, 20]; thus, our infection rate is comparable to those in the literatures. Four hundred fifty-one cases (66.2%, 451/681) of calcaneus fractures were treated by ORIF via minimally invasive approaches (the limited lateral, obtuse-angled, medial, combined medial and lateral, posterior, percutaneous, and sinus tarsi approaches). Although the investigation of this technique is still ongoing and controversial [20, 23, 24], minimally invasive techniques minimize wound-related complications. The high proportion of minimally invasive approaches in our study likely contributes to a lower incidence of superficial wound infection compared to other investigations [6, 25].

Open fracture as a significant risk factor for postoperative SSI after traumatic fractures of calcaneus and other disciplines has been investigated in literatures [11, 22, 26, 27]. There were 5.1% (35/681) open fractures in this study, and patients were 9.48 times as likely to develop an infection after operative treatment (the odds ratio was 10.04, and 9.48 in univariate and multivariate analysis models). Open calcaneus fracture generally was accompanied by contaminative wound, severe soft-tissue damage. In order to preserve a healthy soft-tissue envelope before final fixation, surgeons have traditionally delayed operative treatment for a few days until hematoma eliminated, stress of incision alleviated, and skin wrinkled. However, many controversies surround the benefits of delaying operative fixation of calcaneus fractures. In Nicholas A. Abidi’ study [17], they demonstrated delaying operation at an average of 10 days after injury increased the incidence of wound healing approximately 2.6 times when contrasted with 4.8 days. Preoperative stay was not an independent risk factors in this study (*P* = 0.36943). However, once this interval exceeds 14 days, the incidence of SSI was significantly increased (15.8 versus 9.5%) and the average time between injury occurrence and surgery was 5.6 days (range 0–27 days) in open fracture group. According to our definition of polytrauma, the 35 patients who were diagnosed as open calcaneus fractures sustained a total of 55 traumatic injuries associated with musculoskeletal, cardiothoracic, and other systems. These patients potentially required extra numbers of surgical operations to recuperate their health. Abdo Bachoura and his colleagues [28] identified 1611 adult patients who underwent 1783 surgical procedures for skeletal trauma, and they found multiple surgeries doubling the occurrence of surgical site infection. Our finding highlighted the fact that open fracture, the independent predictor of postoperative SSI, is still an austere challenge to the trauma orthopedic surgeon. Of the 357 high-energy injury, 47 (13.2%) patients suffered from postoperative SSI and 25 cases (7.0%) were open calcaneus fractures. Higher grade fractures usually accompanied by high-energy injury, and this type of fracture had significantly higher wound complication rates than low-grade ones [29].

Open reduction and internal fixation are the optimum strategies for severely displaced and depressed intra-articular or open calcaneus fractures. Although external fixation cannot replace open treatment in calcaneus fractures, it plays an important role in the recovery of the soft tissue. When performed in a stepwise fashion, external fixation can successfully restore normal calcaneal height, length, width, and coronal plane alignment.

It is time-consuming to restore the heel height and length, realign the posterior facet of the subtalar joint, and restore mechanical axis of the hindfoot in higher grade of comminuted calcaneus fractures. Fractures caused by high-energy injury had a longer operation time than lower grade fractures (111.3 min versus 103.9 min) in this study. Univariate analysis demonstrated operative duration as an approximately risk factors of SSI (*P* = 0.05660); however, these variables were not associated with wound infection in the multivariable logistic regression model. Prolonged operative time increasing the SSI rate was also been found in other surgical disciplines [30, 31]. High-energy fractures seems to have a positive correlation on the increased postoperative wound infection [12]. This may be due to high proportion of open fracture, disruption of the microcirculation in the soft-tissue envelope of the calcaneus, and prolonged operative time.

American Society of Anesthesiologists (ASA) System, proposed by Saklad [18], has been used since the 1940s as a reliable health evaluation system to characterize operative risk and assess physical status of patients. Nineteen (19/681, 0.28%) patients’ ASA score were more than or equal to 3 and 26.3% (5/19) of them developed SSI after surgery. In the univariate and multivariable analysis model, ASA class 3 or higher was identified as a predictor of SSI and the odds ratio was 3.52 and 3.50, respectively. Manouk Backeset al [8] retrospectively studied 191 patients who were operatively treated for unilateral calcaneus fractures. They found ASA classification other than 1 (*P* = 0.001) showed an increased risk of a postoperative wound infection. The average age of patients in Manouk Backes’ study was 45 years which was significantly older than that in our investigation (42 years). Patients (ASA class 3 or higher) were 2.87 times as likely to develop an infection after high-energy lower extremity fractures according to Ebrahim Paryavi and his colleagues’ report [19]. Our results also support the strong predictive value of the ASA class for infection risk in orthopedic calcaneus fractures.

The World Health Organization published the Guidelines for Safe Surgery and described “maintenance of normothermia during surgery” as 1 of 10 objectives aimed to reduce the incidence of postoperative surgical site infection in 2009 [32]. Agency for Healthcare Research and Quality has established a similar quality metric using a cutoff of 36.0 °C [33]. However, evidence supporting this hypothesis is lacking to our knowledge and many recent studies demonstrated that there are no correlations between hypothermia and SSI in different disciplines. The normal range of the core body temperature is 36.5~37.5 °C, suppression of the body’s thermoregulatory function by anesthetic drugs, and exposure of viscera or limbs to cold environments can result in hypothermia. It has been confirmed that hypothesis (lower than 36.0 °C) has many adverse effects on the surgical patients, such as blood coagulopathy, disturbances of immune regulation, arrhythmia, and prolonged recovery time of anesthesia. In the present study, 18.2% of patients had a hypothermia intraoperatively, and 21 of these cases developed SSI. Regardless of how hypothermia was defined, intraoperative temperature dose predicts SSI in calcaneus fracture patients, and careful surveillance of intraoperative hypothermia is of clinical significance and should be considered. There are significant benefits of warming in the prevention of postoperative complications. It is important to warming patients preoperatively and monitor core body temperature intraoperatively, and these measures include the use of warmed fluids, heating blankets, elastic bandage, and so on.

Univariate analysis demonstrated preoperative use of antibiotics, operative duration, coronary heart disease, and preoperative basophilic granulocyte (BAS) count as risk factors of SSI; however, these variables were not associated with increased risk of wound infection in the multivariable logistic regression model. Operative time was a well-identified risk factor of postoperative wound infection; however, the impact of operative duration was dwarfed by other more important risk factors in this study (open fractures, high-energy injury). In our clinical practice, the use of antibiotics before surgery is an indication of open fracture and basophilic granulocyte (BAS) count was related to the situation of injury; therefore, these variables were superseded by more direct factor (open fracture, high-energy injury) in final analysis model. Eleven patients were diagnosed with coronary heart disease with an average age of 50.6 years, and 3 cases developed SSI after surgery. However, the remaining patients (98.4%, 670/681) were much younger than the 11 cases on account of a mean age of 42.2 years. Therefore, we infer that coronary heart disease more likely be identified as a significant risk factor in geratic calcaneus fractures.

There are several limitations in this single-center study. First of all, the retrospective nature of this study inherited an unavoidable selection bias and the quality of the data depended on the accuracy and completeness of electronic medical records, which might be affected more or less. Secondly, patient-specific covariates (smoking and alcohol consumption, drugs addict, medical comorbidities,) mainly relied on the patient’s self-report, and some of them might not be willing to inform us of these bad habits and coexisting diseases. Thirdly, we defined a patient who did not return for treatment of an infection after discharge from our trauma center as non-SSI one; therefore, those who truly developed SSI and admitted to anther hospital for diagnosis and treatment could be miss-collected, which might underestimate the overall SSI rate.

## Conclusion

We conduct a retrospective study of consecutive patients with calcaneus fractures treated by ORIF. There were 66 cases developed SSI with an incidence of 9.7% (66/681). The prevalence of superficial and deep infection was 6.8 and 2.9%, respectively. Open fracture, high-energy injury, ASA classification higher than 3, and intraoperative hypothermia were identified as predictors of postoperative SSI. It deserve orthopedic surgeons’ attention of the significance of external fixation and prevention of hypothermia in the treatment of calcaneus fractures. More skilled surgical techniques and more reasonable management strategies on open fracture should be utilized to reduce the SSI development.

## Abbreviations

A/G: Albumin/globulin value
ALB: Albumin
ASA: American Society of Anesthesiologists
BAS: Basophilic granulocyte
BMI: Body mass index
CDC: Center for Disease Control
DIACFs: Displaced intra-articular calcaneus fractures
GLOB: Globulin
LYM: Lymphocyte
MON: Monocyte
NEUT: Neutrophile granulocyte
ORIF: Open reduction and internal fixation
SSI: Surgical site infection
TP: Serum total protein
WBC: White blood cell
